# Sodium-glucose Cotransporter 2 Inhibitors Association with Risk of Heart Failure Hospitalization in Preserved and Mildly Reduced Ejection Fraction, Regardless of Diabetes Mellitus: A Systematic Review and Meta-Analysis

**DOI:** 10.2174/011573403X351298250717031928

**Published:** 2025-07-31

**Authors:** Ayushi Mendiratta, Akshat Banga, Piyush Garg, Vikas Bansal, Jordan Klaassen, Raja Avnesh Reddy, Abubakar Nazir, John Abdel Sayed, Douglas Duffee

**Affiliations:** 1 Department of Internal Medicine, UCHealth Parkview Medical Center, Pueblo, CO81003, USA;; 2 Department of Internal Medicine, Mount Auburn Hospital, Harvard Medical School, Cambridge, MA02138, USA;; 3 Department of Anesthesiology, Fortis Escorts Heart Institute, New Delhi, India;; 4 Department of Research, WellSpan York Hospital, York, PA17403, USA;; 5 Loma Linda University School of Medicine, Loma Linda, California, CA92350, USA;; 6 Department of Internal Medicine, Sinai Hospital of Baltimore, Baltimore, Maryland, MD21215, USA;; 7 King Edward Medical University, Lahore, Pakistan

**Keywords:** Sodium-glucose cotransporter 2 Inhibitors, heart failure with preserved ejection fraction, heart failure with mildly reduced ejection fraction, heart failure hospitalization, heart failure, all-cause mortality

## Abstract

**Introduction:**

There are strong guidelines regarding the importance of SGLT-2 inhibitors (SGLT2i) in reducing mortality in patients with heart failure with reduced ejection (HFrEF). However, the role of SGLT2i in the management of patients with heart failure with preserved ejection fraction (HFpEF) and heart failure with mildly reduced ejection fraction (HFmrEF) remains ambiguous.

**Methods:**

A systematic review and meta-analysis of SGLT2i randomized controlled trials (RCTs) in HFpEF and HFmrEF, with and without diabetes was conducted (Prospero ID - CRD42023464479). Databases including Clinicaltrials.gov, PubMed, Biomed Central, Scopus, and Science Direct were searched from 2018 to 2024. Hospitalization due to heart failure (HFH) with HFpEF and HFmrEF was the primary outcome analyzed, followed by a subgroup analysis of HFH in HFpEF only. Secondary outcomes analyzed included cardiovascular (CV) death, all-cause mortality, and serious adverse effects.

**Results:**

In seven RCTs involving 31,057 participants, meta-analysis using random effects models showed that SGLT2i treated patients had a statistically significant reduction in HFH risk (OR=0.74, *p<*0.00001) compared to placebo or standard of care (SOC). A subgroup analysis, in HFpEF only patients, also showed a statistically significant reduction (OR=0.72, *p<*0.0001) in HFH odds. Statistical analysis of secondary outcomes showed a statistically non-significant difference in CV death risk (OR=0.92, *p=*0.13), all-cause mortality (OR=0.94, *p=*0.13), and any serious adverse events (OR=0.92, *p=*0.10).

**Discussion:**

This meta-analysis demonstrates that SGLT2i significantly reduce the risk of heart failure hospitalization in patients with preserved and mildly reduced ejection fraction, regardless of diabetes status. While reductions in cardiovascular and all-cause mortality, as well as serious adverse events, were observed, these did not reach statistical significance. These findings align with emerging evidence suggesting a broader cardioprotective role for SGLT2i across the heart failure spectrum, although further studies are needed to clarify their mortality benefit and long-term safety in HFpEF and HFmrEF populations.

**Conclusion:**

This meta-analysis found a significant reduction in HFH with the use of SGLT2i in patients with HFpEF and HFmrEF. Secondarily, there was a statistically non-significant reduction in all-cause mortality, CV death risk, and serious adverse events with the use of SGLT2i.

## 1 INTRODUCTION

Heart failure (HF) is a complex and potentially fatal condition marked by morbidity, mortality, and considerable financial burden, impacting over 64 million individuals globally [1]. HF incidence ranges from 1-3% among adults in developed countries, and it is projected to rise substantially due to the advancement in life-extending diagnostic and treatment modalities [1]. According to the 2017-2020 National Health and Nutrition Examination Survey (NHANES), nearly 6.7 million Americans over 20 years of age were diagnosed with HF, increasing from 6.0 million reported by NHANES from 2015 to 2018 [2]. Around half of these individuals, approximately 3.25 million, are believed to have HFpEF [3].

HFpEF arises from a maladaptive cardiac remodeling response to diverse factors, commonly including age, high blood pressure, obesity, and diabetes [3, 4]. Survival for patients with HFpEF is reduced, as evidenced by a reported 5-year mortality rate exceeding 50% [5, 6].

Until now, standard cardiovascular therapies have had minimal impact on HFpEF morbidity and mortality outcomes [5, 7]. Thus, the focus of HFpEF management currently lies in addressing symptoms and managing comorbid conditions [7, 8]. Pharmacological interventions like angiotensin-converting enzyme inhibitors (ACEIs) and angiotensin receptor blockers (ARBs) have demonstrated efficacy in reducing mortality and hospitalization rates among patients with HFrEF but have not shown similar benefits in clinical trials involving HFpEF [8, 9]. Additionally, the efficacy of β-blockade in HFpEF remains largely uncertain. Thus, patients with HFpEF present a unique management subset compared to those with HFrEF [8, 9]. Although primarily an anti-diabetic class of drugs, SGLT2 inhibitors (SGLT2i) have demonstrated unique cardiovascular pleiotropic effects in addition to their anti-hyperglycemic properties [10, 11], which has resulted in a rise in usage, beginning with the fortuitous finding of heart failure hospitalization (HFH) reduction in large SGLT2i safety and efficacy trials [8, 12].

Clear guidelines exist for the use of SGLT2i in HFrEF independent of the presence of type 2 diabetes mellitus which is in contradistinction to HFpEF, although expert consensus is trending in that direction [11, 13-16]. Ongoing clinical studies are further assessing the effects of SGLT2i on clinical outcomes in HF across the spectrum of ejection fraction, including HFmrEF [17]. Although many have reported a decrease in HFH among SGLT2i-treated HFpEF patients, there is no data representing the pooled effect of these drugs in HFpEF. This meta-analysis aims to address this gap.

## 2 METHODOLOGY

This study adhered to the Preferred Reporting Items for Systematic Reviews and Meta-Analyses (PRISMA) criteria [18]. The protocol was registered on PROSPERO (CRD42023464479).

### 2.1 Data Sources and Literature Search Strategy

Two independent researchers (A.M. and D.D.) performed a systematic search for pertinent articles from December 2018 to April 2024, utilizing databases including ClinicalTrials.gov, PubMed, Scopus, ScienceDirect, and BioMed Central (refer to Supplementary Table **1**). A confluence of keywords, such as “SGLT2 inhibitor,” “SGLT2i,” “dapagliflozin,” “empagliflozin,” “sotagliflozin,” “ertugliflozin,” “heart failure,” “heart failure with preserved ejection fraction,” “heart failure with mildly reduced ejection fraction,” “preserved ejection fraction,” “mildly reduced ejection fraction,” “HFpEF,” and “HFmrEF,” were searched electronically [19].

### 2.2 Study Selection and Inclusion Criteria

To ensure accurate study selection and inclusion, duplicate articles from the systematic search were identified and removed using Endnote (Clarivate Analytics, Thomson Reuters Corporation, Philadelphia, Pennsylvania). Two independent researchers (A.M. and A.B.) initially screened the articles based on titles, abstracts, and keywords. Articles deemed relevant were then further evaluated through full-text screening. Data review and collection were conducted by all authors. References from the shortlisted articles were also reviewed to identify additional studies. Any disagreements were resolved through group discussion or by consulting an independent reviewer (V.B.). Articles that did not meet the inclusion criteria were excluded. The inclusion criteria for the meta-analysis were as follows:

1. RCTs involving patients aged 18 years and older.
2. Research comparing results of patients with HFpEF and/or HFmrEF treated with SGLT2i to those receiving a placebo or conventional therapy.
3. Studies presenting data (raw or adjusted) on at least one of the outcomes of interest.

The exclusion criteria consisted of:

1. Studies without a control group.
2. Studies without an outcome of interest (hospitalization due to HF, all-cause mortality, death due to CV cause, severe adverse effect).
3. Narrative reviews, case reports, book chapters, and conference abstracts.

Across the included studies, heart failure diagnosis was based on a consistent set of criteria combining left ventricular ejection fraction (LVEF), elevated NT-proBNP levels, and clinical symptoms. Elevated NT-proBNP served as a central inclusion criterion, with thresholds adjusted based on the presence of atrial fibrillation. Clinical symptoms were defined by New York Heart Association (NYHA) functional classes II–IV, which were consistently required across trials to ensure symptomatic heart failure. Additionally, several studies incorporated structural heart disease markers, including left atrial enlargement or left ventricular hypertrophy, to further refine patient selection. This standardized diagnostic approach ensured alignment in identifying patients with HFpEF/HFmrEF across studies.

### 2.3 Sample Size

The sample size for this meta-analysis was determined by the total number of participants across the included RCTs (n=31,057). No additional power calculation was required, as the study design inherently aggregated available data from all eligible trials.

### 2.4 Outcomes of Interest

The principal outcome of interest was the frequency of HFH in patients with HFpEF and/or HFmrEF using SGLT2i, compared to those receiving placebo, standard-of-care (SOC), or other pharmacological treatments. Cardiovascular mortality, all-cause mortality, and serious adverse effects were considered as secondary outcomes. Both raw data and adjusted estimates were used in the meta-analysis. Estimates from studies that applied propensity-matched scoring were considered adjusted.

### 2.5 Statistical Analysis

We conducted unadjusted and adjusted analyses using Review Manager v.5.4 (The Nordic Cochrane Centre, The Cochrane Collaboration, 2014) [20] for unadjusted and adjusted odds ratios (OR) and Comprehensive Meta-Analysis software package 154 (Biostat, Englewood, NJ, USA) for the meta-regression analysis [21]. Unadjusted analyses were performed using Mantel-Haenszel crude ORs with a 95% confidence interval (CI) in a random-effects model. Adjusted odds ratios (aOR’s) with a 95% CI were calculated to account for baseline differences, providing more robust primary analysis results, particularly in comparing SGLT2i with placebo/SOC. Statistical significance was defined as *p<*0.05, and all analyses applied a random-effects model. Heterogeneity was assessed with Higgins’ I^2^, with I^2^ > 50% indicating substantial heterogeneity [22].

To reduce selection bias inherent in RCTs, subgroup analyses based on study design were performed [22]. We also conducted univariate and multivariate meta-regression analyses with random effects to explore study-level variability. Potential sources of variability included age, gender, country, and prevalence of comorbid conditions (*e.g.*, obesity, diabetes, dyslipidemia, cardiovascular disease, hypertension, and mean LVEF). Covariates with a significant effect (*p<*0.05) on hospitalization, mortality, or adverse outcomes in HFpEF or HFmrEF patients treated with SGLT2 inhibitors were included in further modeling. Two models—one for mortality and one for condition severity—were developed. Preselected factors were incorporated into a manual backward, stepwise multiple meta-regression analysis, with *p<*0.05 as the threshold for statistical significance. All tests were two-tailed.

### 2.6 Risk of Bias and Quality Assessment

A.M. and D.D. independently assessed the included studies’ methodological quality through the Cochrane Risk Of Bias tool and the correlation of quality measures with estimates of treatment effects in the meta-analysis of RCTs [23, 24]. The studies were evaluated based on the following criteria: (i) Selection of the study group, (ii) Comparability of the study groups, and (iii) Assessment of the desired outcomes. Any disagreements were resolved through discussion with a third investigator (A.B.). To assess the likelihood of publication bias, Egger regression methods were applied, and the results were visually inspected using funnel plots. The certainty of evidence at the outcome level was determined using the GRADE pro profiler (GRADE working group, McMaster University, and Evidence Prime Inc.) [25].

## 3 RESULTS

### 3.1 Literature Search

**Fig. (1):**
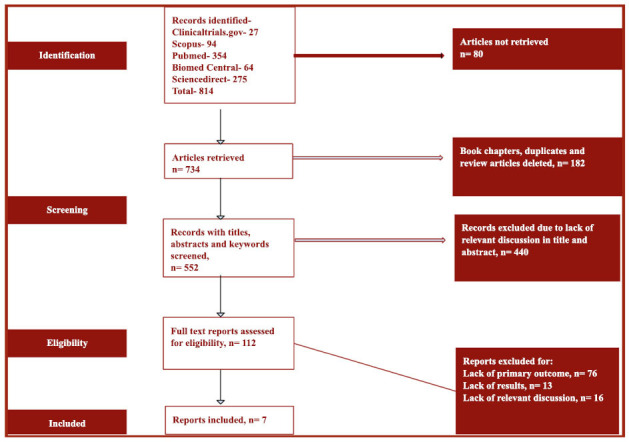
PRISMA flow diagram.

A total of 814 articles matched the inclusion criteria during the initial database search on PubMed, Cochrane Library, Science Direct, and Web of Science. Of these, 80 articles were not retrieved. From the remaining 734 articles, 182 duplicates, narrative review articles, and book chapters were identified and removed before screening. The remaining 552 articles were screened by two authors independently, and 440 were excluded due to a lack of relevance in the title, abstract, and keywords. Therefore, 112 articles were sought for full-text review. Eventually, 7 articles that met the eligibility criteria of our study were included in the analysis (Fig. **1**).

### 3.2 Study Characteristics

Our meta-analysis assessed data from 31,057 patients across seven RCTs published between 2018 and 2024. Of these, 15,708 cases were treated with an SGLT2i, and 15,349 controls were managed with a placebo or the standard of care (SOC), except for one RCT in which controls were treated with glimepiride. Three RCTs reported the use of dapagliflozin, while canagliflozin was used by two RCTs, and the remaining two RCTs reported empagliflozin and ertugliflozin respectively. The study mean interval period ranged from three to 50.4 months. All seven RCTs reported hospitalization and all-cause mortality data, five RCTs reported cardiovascular mortality and any severe adverse effects [26-32] respectively.

Dosing protocols for SGLT2 inhibitors were generally harmonized across trials, with most studies using standard doses. The VERTIS CV trial evaluated ertugliflozin at both 5 mg and 15 mg doses to explore dose-specific effects [27]. Across studies, no dose adjustments were made for renal function, as patients with severe renal impairment (eGFR <25 mL/min) were excluded, minimizing the need for renal-based dosing modifications. The main characteristics of the studies included are summarized in Table **1**.

### 3.3 Primary Outcome: Effect of SGLT2i on Hospitalization due to HF

All seven studies evaluated the risk of hospitalization using SGLT2i [26-32]. Using the random effects model, SGLT2i showed a statistically significant reduction in risk of HFH (OR=0.74, 95% CI 0.67-0.81, *p<*0.00001, I^2^=0%), indicating a 26% reduction in odds. This suggests that despite differences in study characteristics, there is a consistent benefit across the trials (Fig. **2a**). The Egger’s test results did not show statistically significant evidence of publication bias (*p=*0.98). No major differences between published and unpublished studies were observed. However, smaller studies might have overestimated the effect size (Supplementary Fig. **1a**).

#### 3.3.1 After Excluding LVEF <50%

Further subgroup analysis on HFH in patients with preserved EF was performed after excluding studies that commingled LVEF<50% using the random effects model. This showed a statistically significant reduction in HFH risk due to HFpEF (OR=0.72, 95% CI 0.61-0.85, *p<*0.0001, I^2^=0%), indicating a 28% reduction in odds of heart failure hospitalization (Fig. **2b**). The Egger's test results did not yield statistically significant evidence of publication bias (*p=*0.87). However, the test's ability to detect true publication bias is considerably reduced with such a small sample size (Supplementary Fig. **1b**).

### 3.4 Secondary Outcomes

Secondary outcomes studied in this meta-analysis were death due to CV causes, all-cause mortality, and any serious adverse events in the applicable studies.

#### 3.4.1 Death Due to Cardiovascular Causes

Five studies reported death due to CV causes in this meta-analysis [26, 27, 29, 31, 32]. Using the random effects model, there was a statistically non-significant reduction in risk (OR=0.92, 95% CI 0.83-1.02, *p=*0.13, I^2^=0%) of death due to CV causes in the SGLT2i group (Fig. **3a**). Egger's test results did not show statistically significant evidence of publication bias (*p=*0.73) (Supplementary Fig. **2**). The limited number of studies reduces the test's power to definitively detect publication bias and indicates a trend of overestimating the effect.

#### 3.4.2 All-cause Mortality

All-cause mortality was studied in all seven RCTs [26-32]. Using the random effects model, there was a statistically non-significant reduction (OR=0.94, 95% CI 0.87, 1.02, *p=*0.13, I^2^=0%) in all-cause mortality risk with SGLT2i use when compared to those treated with a placebo or the standard of care (Fig. **3b**). Egger's test results did not show statistically significant evidence of publication bias (*p=*0.88) (Supplementary Fig. **3**). A larger number of studies led to a higher sensitivity of the test towards detecting bias. No clear trends were observed between the study size and effect size.

##### 3.4.2.1 Serious Adverse Effects

Serious adverse events were reported in five studies [28-32]. Using the random effects model, there was a statistically non-significant decrease in risk (OR=0.92, 95% CI 0.83-1.02, *p=*0.10, I^2^=35%) of any serious adverse events in the groups receiving SGLT2i compared to the groups not receiving them (Fig. **4a**). The Egger's test results did not show statistically significant evidence of publication bias (*p=*0.19) (Supplementary Fig. **4**). The non-significant p-value reinforces the uncertainty surrounding this observation, as the finding was surprising given well known SGLT2i side effects such as amputation, genital infection, and euglycemic DKA.

##### 3.4.2.2 Sensitivity Analysis for any Serious Adverse Events after Excluding Nassif *et al.* (2021)

In light of the adverse event findings summarized in Fig. (**4a**) above, additional analysis was conducted excluding Nassif *et al*., as it appeared to be an outlier. Using the random effects model, there was a statistically significant difference in risk (OR=0.91, 95% CI 0.85-0.98, *p=*0.007, I^2^=13%) of any serious adverse events in the groups receiving SGLT2i compared to the groups not receiving them (Fig. **4b**). Given the differences in the reporting of SAE’s in previous clinical SGLT2i trials, we suspect better standardization of adverse event monitoring will help to clarify these results.

### 3.5 Risk of Bias

The Cochrane tool for risk of bias was used for RCTs (Supplementary Table **2a**) [23], and the correlation of quality measures with estimates of treatment effects in meta-analyses of RCTs [24] was used for the quality assessment of the same (Supplementary Table **2b**). Quality assessments were conducted independently, and discrepancies were resolved by consensus. Overall, the risk-of-bias assessment showed that the included studies had a low to high risk of bias. (Supplementari Figs. **1-4**) illustrate the funnel plots assessing publication bias, finding none. Additionally, these figures present various p-values derived from Egger's regression tests. We assessed the certainty of evidence at the outcome level using the GRADE pro profiler (GRADE working group, McMaster University, and Evidence Prime Inc.) (Supplementary Table **3**) [25]. All the outcomes, including HFH, HFH after excluding LVEF <50%, cardiovascular mortality, all-cause mortality, and severe adverse effects, had a moderate grade of evidence certainty.

## 4 DISCUSSION

The current systematic review and meta-analysis of seven RCTs and 31,057 patients with HFpEF and HFmrEF, from December 2018 to April 2024, provides a comprehensive assessment of the association of SGLT2is with HFH, CV death, all-cause mortality, and severe adverse effects in HF patients with preserved and mildly reduced EF. Our results showed a significant impact of SGLT2i in reducing HFH risk by 26% among adult subjects compared to placebo or SOC. SGLT2i use was also found to have a positive, yet statistically insignificant, effect on secondary outcomes, reducing CV and all-cause mortality and major adverse effects in our unadjusted analysis.

A significant benefit in hospitalization outcomes has been observed in the EMPEROR-Preserved, DECLARE–TIMI, CANDLE, VERTIS CV, DELIVER, and CANONICAL clinical trials, except for the PRESERVED-HF trial. While most included trials focused on chronic heart failure populations, DELIVER and VERTIS CV enrolled both newly diagnosed and chronic HF cases but did not differentiate outcomes based on HF duration. This heterogeneity in patient populations may influence treatment response and should be considered when interpreting the results. Conversely, none of the trials examining the benefits of SGLT2is in reducing CV mortality [26, 27, 29, 31, 32], all-cause mortality [26-32], and the risk of severe adverse effects [28-32] found a significant effect, either individually or in pooled estimates.

SGLT2i are a class of anti-diabetic drugs that act upon the SGLT-2 receptors on the intestine and kidney to prevent the absorption and reabsorption of glucose and sodium from the intestine and proximal tubules of the kidneys, respectively, thus increasing the excretion of glucose and sodium [33]. SGLT2i have demonstrated pleiotropic effects on various molecular and physiological systems, including the CV system [34]. Several hypotheses regarding direct and indirect mechanisms underlying the cardiometabolic benefits of SGLT2i have been proposed. These include augmented natriuresis and osmotic diuresis, improved calcium regulation, elimination of glucose in the urine, glycemic control, blood pressure reduction, weight loss, decreased intraglomerular pressure, protection of the cardiorenal axis, and decreased inflammation through various pathways, including those originating from epicardial fat [35, 36]. A study by Lopaschuk *et al*. [37] explained the cardioprotective effect of SGLT2i through a reduction in oxidative stress and the prevention of cardiac glucotoxicity. Another study by Graffin *et al*. [38] showed that empagliflozin effectively increased natriuresis and reduced plasma volume in patients with T2DM and stable HF. DeSa *et al*. [39] stated that by decreasing blood glucose levels, SGLT2i may indirectly control the cardiotoxic effect of hyperglycemia. However, the specific mechanisms responsible for the reduction in atherosclerotic events, HFH, and the increased Kansas City Cardiomyopathy Questionnaire Clinical Summary Score (KCCQ-CSS) remain debatable and indicate the need for further research.

The role of SGLT2i’s within guideline-directed medical therapy for symptomatic heart failure with reduced EF is well established [11]. How the effectiveness of SGLT2i therapy extends to other forms of heart failure, in particular HFpEF and HFmrEF, in terms of HFH rates and mortality in these patients remains questionable. A meta-analysis by Cardoso *et al*. found a composite of CV mortality, HFH, or urgent visits for HF to be significantly lower in the HF population of combined HFrEF and HFpEF treated with SGLT2i [40]. They also reported a reduction in CV death and HFH in HFpEF patients using SGLT2i in their secondary analysis [40]. These results are supported by Butler *et al.’s* [41] meta-analysis, where the risks of first HFH and a composite of HFH or CV death were significantly lower in the SGLT2i-treated HFpEF patients compared to the controls. In contrast, CV death and all-cause mortality were comparable between the two groups [41]. Similarly, recent meta-analyses by Jaiswal *et al*. and Zhou *et al*. emphasized that SGLT2i markedly decreased the occurrence of initial HFH and the composite outcome of CVD mortality or HFH in patients with HFpEF [17, 42]. However, CV and all-cause mortality were comparable between both groups. These findings are partially consistent with our primary outcome, which shows a reduction in HFH in patients treated with SGLT2i for HFpEF and HFmrEF.

Multiple outcomes trials focusing on SGLT2i, such as dapagliflozin, empagliflozin, and ertugliflozin, primarily targeted HFH and CV death in HFpEF patients [26, 27, 29]. Additionally, the DECLARE-TIMI trial considered MACE as a primary outcome parameter, alongside HFH and CV death [32]. The reduction in HFH-related events appears to significantly contribute to the composite outcome of total HFH or CV death events in these trials. Conversely, the PRESERVED-HF trial measured the KCCQ-CS as the primary outcome and reported improvements in symptoms, physical limitations, and exercise function in HFpEF patients treated with the SGLT2i dapagliflozin [28]. Alternatively, the CANONICAL and CANDLE trials primarily assessed the benefits of the SGLT2i canagliflozin on body weight and plasma B-type natriuretic peptide (BNP) concentrations, reporting significant and non-significant improvements in body weight and plasma BNP, respectively [30, 31].

In our study, four SGLT2i were studied: dapagliflozin, empagliflozin, canagliflozin, and ertugliflozin. All these agents specifically target SGLT2 receptors, while another drug within the same class, sotagliflozin, is a dual SGLT2 and SGLT1 inhibitor. All five SGLT2i are US FDA-approved in adults with HF or T2D, chronic renal disease, and other CV risk factors [43-45]. SGLT2i have demonstrated notable therapeutic benefits in conditions like DM, HF, and CKD, yet their usage is accompanied by potential adverse effects. These include acute kidney injury (AKI), diabetic ketoacidosis (DKA), urogenital infections including infrequent yet severe diseases such as Fournier's gangrene, bone fractures, and lower limb amputations [46]. For instance, increased risks of urinary tract infections (UTIs) and DKA were highlighted by the U.S. Food and Drug Administration (FDA) in 2015, and reports of AKI cases led to FDA safety communications in 2016 [47]. Although some meta-analyses have not found significant increases in fracture or UTI risk, concerns persist regarding the potential occurrence of these events, along with hypoglycemia and hypotension [47].

Among SGLT2 inhibitors, canagliflozin has been uniquely associated with an increased risk of lower limb amputations, as reported in the CANVAS trial, where the amputation risk was nearly doubled in the canagliflozin group compared to the placebo [48]. In contrast, empagliflozin, dapagliflozin, and ertugliflozin have not demonstrated an elevated amputation risk in major trials such as EMPA-REG OUTCOME, DECLARE-TIMI 58, and VERTIS CV [27, 32, 49]. Observational studies have yielded conflicting results, with some confirming an increased amputation risk, while others found no significant correlation after adjusting for confounders [50-52]. Due to these findings, regulatory bodies, including the FDA and the European Medicines Agency (EMA), have advised caution when prescribing canagliflozin, particularly in patients with a history of amputations, peripheral artery disease, diabetic neuropathy, or poor glycemic control [53].

Despite these concerns, overall, SGLT2i maintain an acceptable safety profile, as seen in our study, underscoring the importance of careful patient monitoring and risk assessment when prescribing these medications.

### 4.1 Clinical Implications

These findings in patients with “non-reduced EF” HF highlight important implications regarding the scope of use of SGLT2i therapy. The observation that lesser forms of LV dysfunction benefit from SGLT2i therapy supports building awareness for SGLT2i use overall, with a willingness to initiate SGLT2i therapy in patients with both full and subtle clinical HF and preserved or moderately reduced EF. It also raises the potential consideration for earlier treatment initiation in these patients prior to developing full clinical HF, especially in those who may be categorized as stage B HF from a diastolic perspective.

Knowing that at-risk hearts with and without diabetes benefit from SGLT2i therapy further extends the use of SGLT2i therapy from the endocrinologic to the CV domain. However, the optimal timing for initiation, identification of high-risk individuals, and the most effective biomarkers for guiding therapy remain areas requiring further investigation [54]. Notably, RCTs focusing on early-stage HF or pre-HF populations (Stage A/B HF) are necessary to evaluate the preventive potential of SGLT2i in halting disease progression in high-risk populations.

Furthermore, given the non-significant impact on mortality, clinicians should be mindful of this limitation when considering SGLT2i for HFpEF treatment. Additional research incorporating individual patient-level data and focusing on more extensive datasets and trials with longer follow-up durations is necessary to understand whether demographic and clinical factors, longer follow-up periods, or combination therapies might influence treatment efficacy and enhance the survival benefits of SGLT2i in this population.

### 4.2 Limitations

The study was limited by the following factors: Some RCTs lacked subgroup data for all outcomes, leading to the exclusion of certain studies from specific endpoint analyses. The statistically non-significant impact of SGLT2i on cardiovascular mortality and all-cause mortality may limit their broader clinical applicability in HFpEF management. However, this could be due to the meta-analysis’s reliance on study-level data rather than individual patient-level data, which restricts our ability to conduct detailed subgroup analyses based on patient characteristics such as age, gender, comorbidity status, or LVEF, limiting a more granular assessment of heart failure subgroups. Additionally, the limited number of studies (<5) analyzing cardiovascular mortality, all-cause mortality, and major adverse events reduces the statistical power of the regression analysis and increases the risk of publication bias.

Variations in follow-up duration across trials (ranging from three to 50 months) may have influenced the observed outcomes. Differences in follow-up lengths could affect the assessment of long-term benefits or risks associated with SGLT2i therapy. While most pooled analyses showed minimal heterogeneity, moderate heterogeneity (I^2^ = 57%) in HF hospitalization data suggests early benefits of SGLT2i treatment. The absence of patient-level data prevents the aggregation of incident numbers and a more precise stratification of subpopulations.

Furthermore, our analysis consolidated all SGLT2i into a single intervention group, preventing a drug-specific evaluation of efficacy. Although pooling data from different SGLT2i agents might obscure drug-specific effects, performing separate analyses for each agent is not feasible due to the limited number of RCTs available for individual drugs. Only three RCTs assessed dapagliflozin, two investigated canagliflozin, and two examined empagliflozin, making individual-agent meta-analyses statistically underpowered. Additionally, all SGLT2i share common mechanisms of action, including natriuresis, osmotic diuresis, and cardiovascular protection, supporting their collective assessment. Finally, inconsistent definitions and reporting of serious adverse events (SAEs) across studies may introduce bias and raise the possibility of underestimating safety risks, warranting cautious interpretation of the safety profile. These limitations may affect the generalizability of our findings, particularly in diverse patient populations and real-world clinical settings

## CONCLUSION

Many RCTs have assessed the correlations between the effectiveness of SGLT2i in HFrEF patients in reducing CV mortality, but only a few studies have included an endpoint of studying their importance in HFH rates in patients with HFpEF and HFmrEF. This meta-analysis confirms that SGLT2i significantly reduce HFH in these populations, reinforcing their role in HF management beyond HFrEF and supporting their broader use across the HF spectrum.

Despite the demonstrated benefits, the impact of SGLT2i on secondary outcomes, including mortality and adverse reactions, remains inconclusive, necessitating longer-term studies with patient-level data to clarify survival benefits. Given the progressive nature of HF, early intervention strategies, particularly in Stage A/B HF, should be prioritized to assess their potential in preventing HF onset and reducing HFH risk. Further stratified analyses are needed to determine whether specific subgroups—such as patients with diabetes, varying degrees of LV dysfunction, or high-risk comorbidities—derive greater benefits from SGLT2i therapy. Expanding clinical trials to evaluate long-term efficacy, safety profiles, and combination strategies with other HF therapies will be crucial in optimizing patient selection and treatment outcomes.

## DECLARATION OF GENERATIVE AI AND AI-ASSISTED TECHNOLOGIES IN THE WRITING PROCESS

During the preparation of this work, the authors used Grammarly to improve the language and grammar of the text. After using this tool/service, the authors reviewed and edited the content as needed and take full responsibility for the content of the publication.

## AUTHORS’ CONTRIBUTIONS

The authors confirm their contribution to the paper as follows: study conception and design: AM and DD; data collection: PG and JK; data curation: RAR and AN; data analysis and interpretation: VB; writing - reviewing and editing: JAS; writing - original draft preparation: AB. All authors reviewed the results and approved the final version of the manuscript.

## Figures and Tables

**Fig. (2a):**
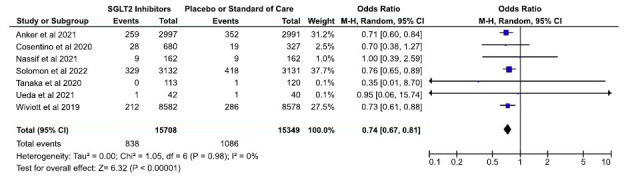
Forest plot risk of heart failure hospitalization in patients, HFpEF and HFmrEF.

**Fig. (2b):**
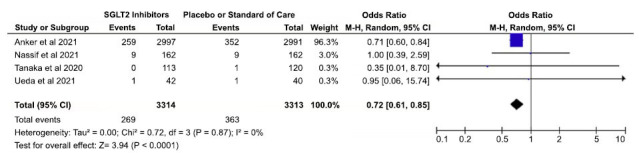
Forest plot subgroup risk of heart failure hospitalization in patients, HfpEF only.

**Fig. (3a):**
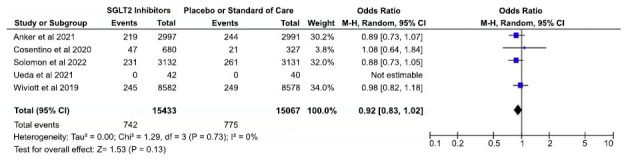
Secondary outcome: Death due to cardiovascular causes.

**Fig. (3b):**
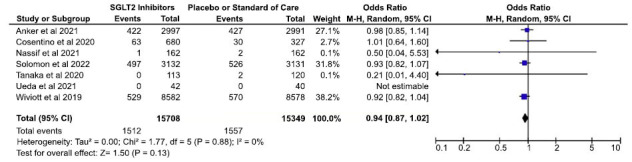
Secondary outcome: All-cause mortality.

**Fig. (4a):**
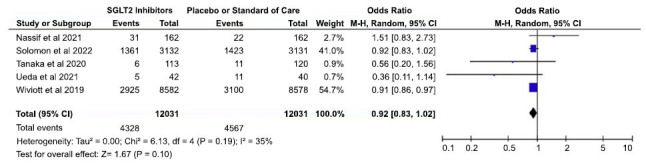
Secondary outcome: Any serious adverse events.

**Fig. (4b):**
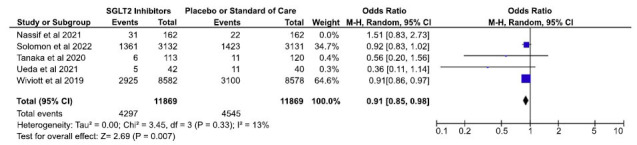
Secondary outcome subgroup: Any serious adverse events after excluding Nassif *et al.* 2021.

**Table 1:** Study characteristics of the included Randomized controlled trials.

**Author**	**Solomon *et al.***		**Nassif *et al.***	**Ueda *et al.***	**Anker *et al.***	**Tanaka *et al*.**	**Consentino *et al.***	**Wiviott *et al.***
Trial Name	DELIVER		PRESERVED-HF trial	CANONICAL	EMPEROR-Preserved trial	CANDLE	VERTIS CV	DECLARE-TIMI
Year of the study	2022		2021	2021	2021	2020	2020	2019
Study design	RCT		RCT	RCT	RCT	RCT	RCT	RCT
Intervention	Dapagliflozin		Dapagliflozin	Canagliflozin	Empagliflozin	Canagliflozin	Ertugliflozin	Dapagliflozin
Sample size – HF (control)	3132		162	40	2991	120	327	8578
Sample size- HF (case)	3131		162	42	2997	113	680	8582
Study period (months)	27.6		50	29	37	26	73	66
Mean intervention period (months)	27.6		3	6	26.2	6	42	50.4
Age (control)		71.5	71 (63, 78)*	75.9	71.9	68.9	64.7	64
SD		9.5	-	5.8	9.6	10.4	8.2	6.8
Age (case)		71.8	69 (64, 77)*	76.5	71.8	68.3	63.8	63.9
SD		9.6	-	6.4	9.3	9.8	8.3	6.8
Gender (control) (M:F)		1749/1383	70/92	27/13	1653/1338	86/34	206/121	5381/3171
Gender (case) (M:F)		1767/1364	70/92	28/14	1659/1338	88/25	446/234	5327/3251
Mean BMI (control)		N/A	34.6	25.2	29.9	25.4	32.9	32
SD		N/A	5	3.7	5.9	4.8	5.3	6.1
Mean BMI (cases)		N/A	35.1	24.7	29.77	68.3	32.6	32.1
SD		N/A	6	3.6	5.8	9.8	5.3	6
Diabetes (control)		1405	91	40	1472	120	327	N/A
Diabetes (cases)		1401	90	42	1466	113	680	N/A
Hypertension (control)		2798	No	34	2703	53	315	N/A
Hypertension (case)		2755	N/A	40	2721	49	639	N/A
LVEF (control)		>40	60±6	61.9±7.6	>50	>50	>45	> 45
LVEF (cases)		>40	60±5	61.1±7.8	>50	>50	>45	> 45

**Abbreviations:** *Median; HF: Heart failure, BMI: Basal metabolic index; LVEF: Left ventricular ejection fraction; SD: Standard deviation; RCT: Randomized controlled trial.

## Data Availability

All data generated or analyzed during this study are included in this published article.

## LIST OF ABBREVIATIONS

HF: Heart Failure
HFmrEF: Heart Failure with Mildly Reduced Ejection Fraction
HFrEF: Heart Failure with Reduced Ejection
LVEF: Left Ventricular Ejection Fraction
NHANES: National Health and Nutrition Examination Survey
RCTs: Randomized Controlled Trials
SGLT2i: Sodium-glucose Cotransporter 2 Inhibitors
SOC: Standard-of-Care
UTIs: Urinary Tract Infections

## STANDARDS OF REPORTING

PRISMA guidelines were followed.

## SUPPLEMENTARY MATERIAL

PRISMA checklist is available as supplementary material on the publisher’s website along with the published article.
