# Supplementary material for: Sodium-glucose Cotransporter 2 Inhibitors Association with Risk of Heart Failure Hospitalization in Preserved and Mildly Reduced Ejection Fraction, Regardless of Diabetes Mellitus: A Systematic Review and Meta-Analysis
Source: Curr Cardiol Rev. 2025 Jul 31;22(3):E1573403X351298. doi: 10.2174/011573403X351298250717031928 (PMC13273753; doi:10.2174/011573403X351298250717031928)
Supplement: Supplementary file 1 [file CCR-22-3-E1573403X351298_SD1.pdf]

Supplementary Material

Sodium-glucose Cotransporter 2 Inhibitors Association with Risk of Heart Failure Hospitalization in Preserved and Mildly Reduced Ejection Fraction, Regardless of Diabetes Mellitus: A Systematic Review and Meta-Analysis

Ayushi Mendiratta[1,*], Akshat Banga[2,*], Piyush Garg[3], Vikas Bansal[4], Jordan Klaassen[5], Raja Avnesh Reddy[6], Abubakar Nazir[7], John Abdel Sayed[1] and Douglas Duffee[1]

[1]*Department of Internal Medicine, UCHealth Parkview Medical Center, Pueblo, CO, USA;* [2]*Department of Internal Medicine, Mount Auburn Hospital, Harvard Medical School, Cambridge, MA, USA;* [3]*Department of Anesthesiology, Fortis Escorts Heart Institute, New Delhi, India;* [4]*Department of Research, WellSpan Hospital, York, PA, USA;* [5]*Medical Student, Loma Linda University School of Medicine, Loma Linda, California, USA;* [6]*Department of Internal Medicine, Sinai Hospital of Baltimore, Baltimore, Maryland, USA;* [7]*Medical Student, King Edward Medical University, Lahore, Pakistan*

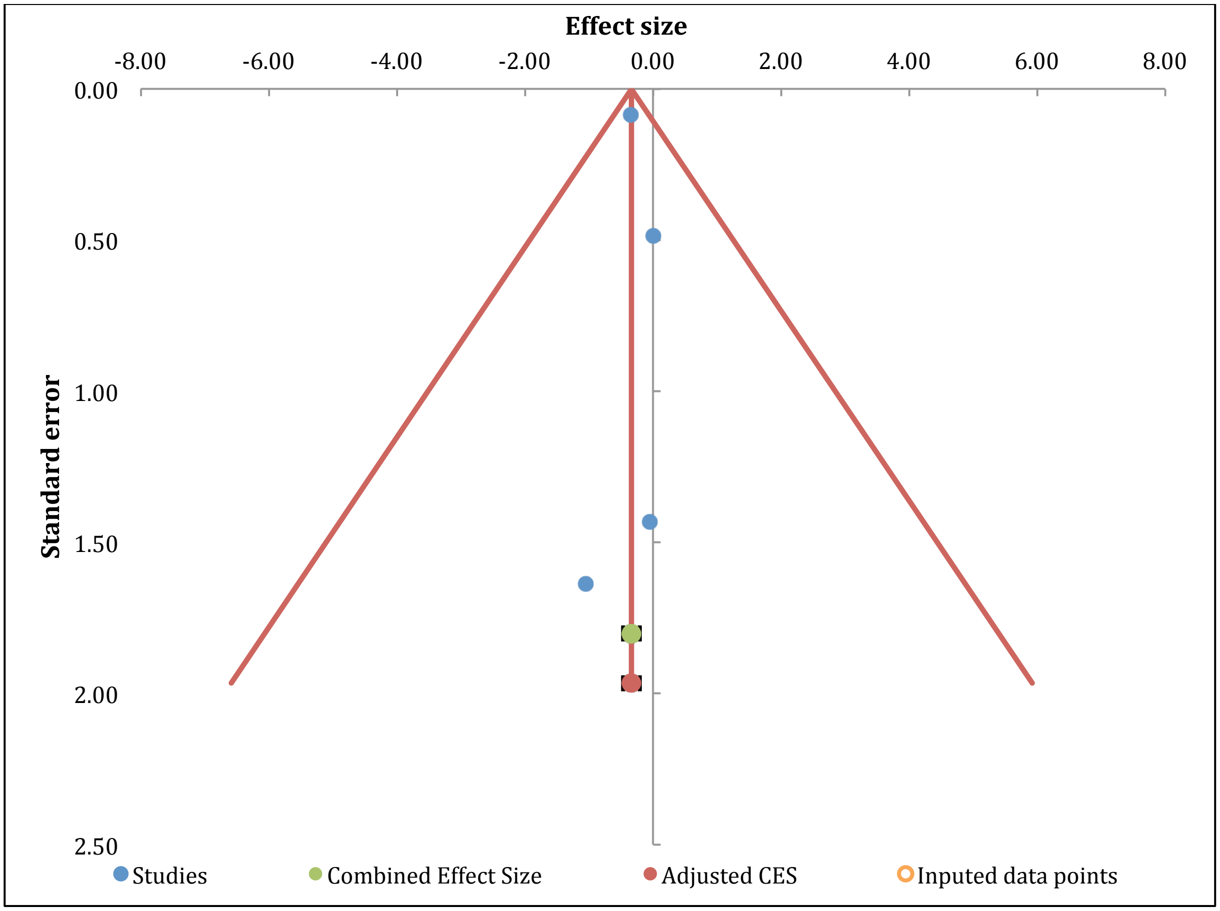

Figure-1a. Funnel plot showing Hospitalization due to Heart Failure

Egger Regression					t test	p-value
	Estimate	SE	CI LL	CI UL		
Intercept	0.03	0.27	-0.63	0.68	0.09	0.929
Slope	-0.31	0.03	-0.39	-0.22		

The funnel plot visually displayed the distribution of effect sizes and study precision. Three studies fell along an adjusted Control Estimator's (CES) line, suggesting minimal bias. Two studies appeared above the funnel plot, potentially indicating an overestimation of the effect. It's important to note that one additional study with a log odds ratio of 0.00 and standard error of 0.49 wasn't included in the visual plot, potentially due to its specific data (a log odds ratio of zero). The Egger's test results did not show statistically significant evidence of publication bias (intercept: 0.03, p-value = 0.929, slope: -0.31, p-value = 0.929). The intercept close to zero and non-significant p-value suggest no major difference between published and unpublished studies. However, the negative slope estimate (-0.31) might indicate a trend towards smaller studies overestimating the effect size. The non-significant p-value associated with the slope (0.929) should be interpreted with caution due to the limited sample size (n = 7). With a small number of studies, the power of Egger's test to detect publication bias is reduced. Therefore, while the results do not definitively point towards publication bias, the limited sample size necessitates cautious interpretation. Future research with larger samples is warranted for a more conclusive assessment.

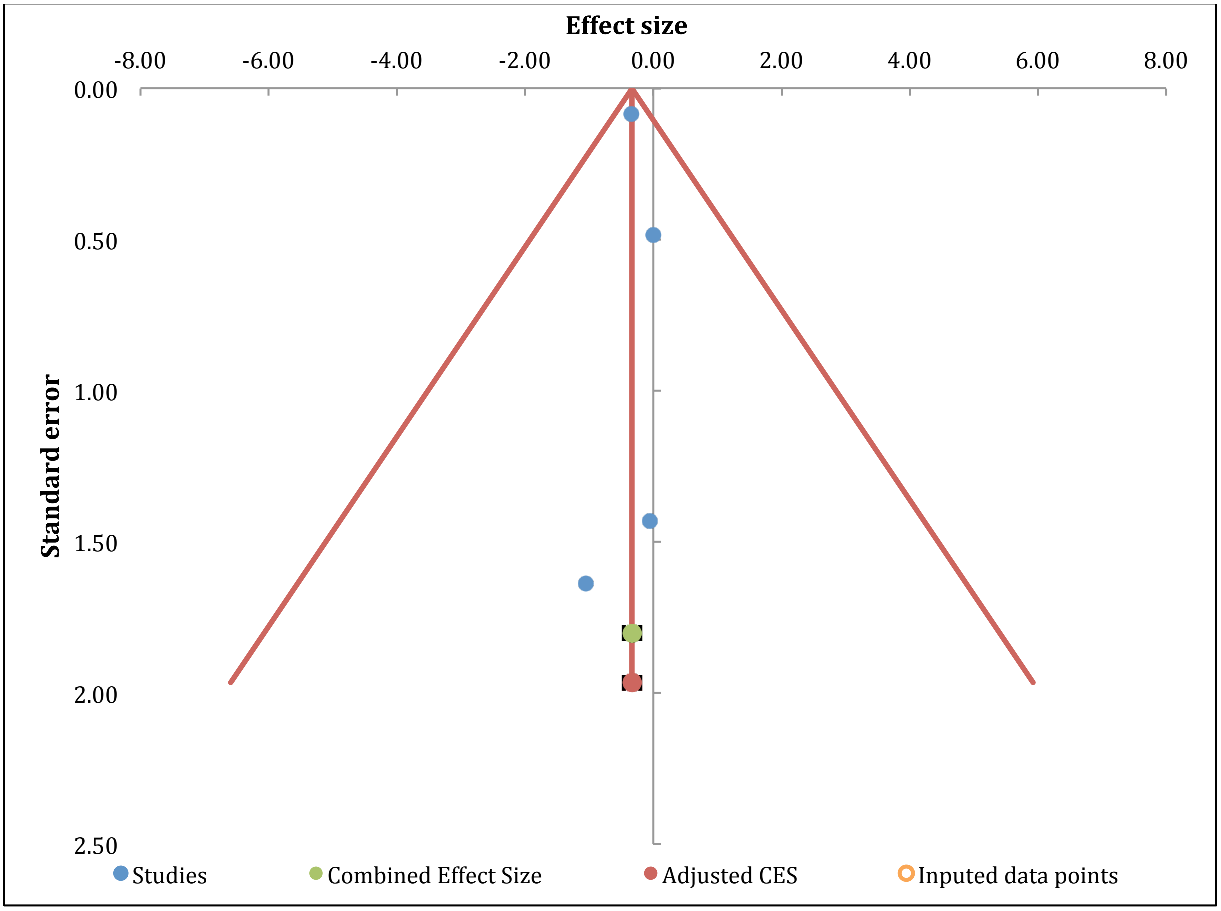

Figure-1b. Subgroup analysis: Hospitalization Due to heart Failure after excluding LvEF <50%

Egger Regression					t test	p-value
	Estimate	SE	CI LL	CI UL		
Intercept	0.14	0.37	-1.05	1.33	0.38	0.740
Slope	-0.35	0.06	-0.55	-0.15		

Due to the small number of studies (n = 4) included in this subgroup analysis, interpreting the Egger's regression test for publication bias is challenging. While the funnel plot visually displayed the distribution of effect sizes and one study appeared above it (potentially indicating an overestimate), drawing conclusions from this limited data is difficult. The Egger's test results themselves did not yield statistically significant evidence of publication bias (intercept: 0.14, p-value = 0.740, slope: -0.35, p-value = 0.740). However, with such a small sample size, the test's ability to detect true publication bias is considerably reduced. Therefore, the possibility of publication bias cannot be definitively ruled out based on this analysis. Future research with a larger number of studies is necessary for a more robust assessment.

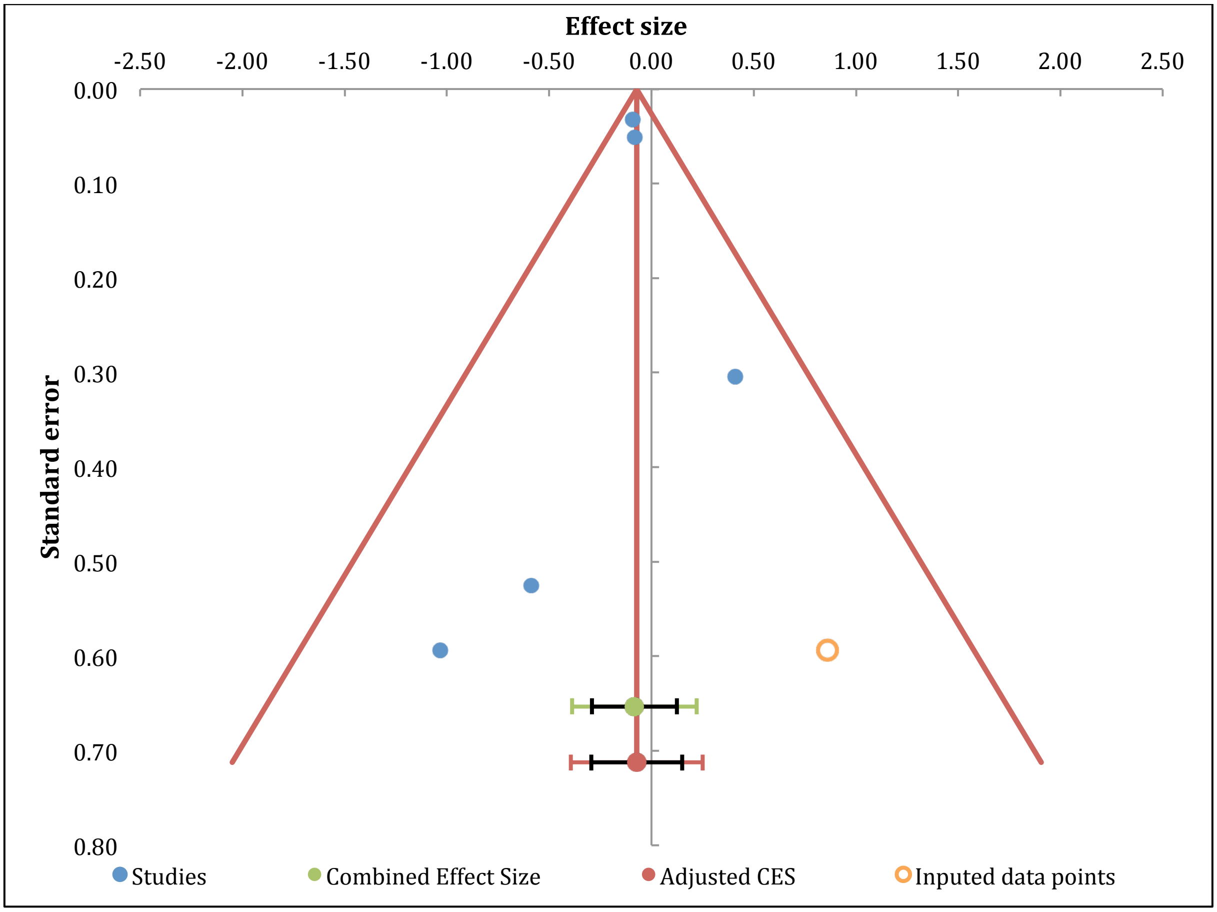

Figure-2. Death due to cardiovascular causes

Egger Regression					t test	p-value
	**Estimate**	**SE**	**CI LL**	**CI UL**		
Intercept	0.28	0.55	-1.25	1.81	0.51	0.646
Slope	-0.11	0.07	-0.29	0.07		

The funnel plot visually depicted the relationship between study size (standard error) and effect size. Four studies fell along an adjusted Control Estimator's (CES) line, suggesting minimal bias. Three studies appeared above the funnel plot, potentially indicating an overestimation of the effect, while one study fell below the line. The Egger's test results themselves didn't show statistically significant evidence of publication bias (intercept: 0.28, p-value = 0.646). However, due to the small sample size (n = 5), caution is necessary. This limited number of studies reduces the test's power to definitively detect publication bias, even if it might exist. The slope of the regression line (-0.11) suggests a very weak, if any, trend where smaller studies might overestimate the effect. However, the p-value associated with the slope (0.646) is not statistically significant, meaning we can't draw strong conclusions from this observation.

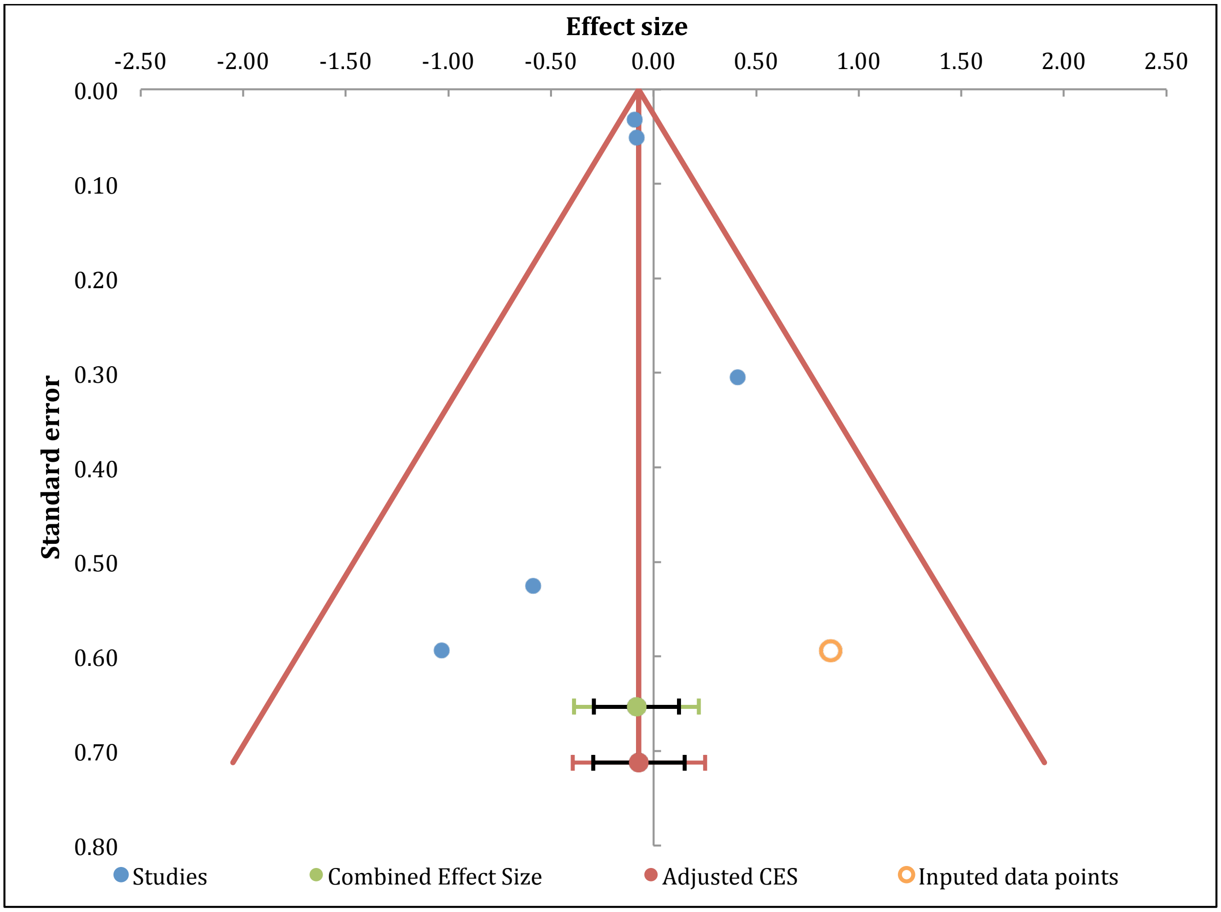

Figure-3. All-cause mortality

Egger Regression					t test	p-value
	Estimate	SE	CI LL	CI UL		
Intercept	-0.35	0.29	-1.06	0.36	-1.20	0.284
Slope	-0.03	0.03	-0.10	0.04		

The funnel plot visually displayed the relationship between study size (standard error) and effect size. Five studies fell along an adjusted Control Estimator's (CES) line, suggesting minimal bias. Three studies appeared above the funnel plot, potentially indicating an overestimation of the effect. One study fell below the line, and one data point with imputed data appeared on the positive side of the plot (represented by a green circle). The Egger's test results themselves didn't show statistically significant evidence of publication bias (intercept: -0.35, p-value = 0.284). However, some caution is necessary due to the still-moderate sample size (n = 7). With a larger number of studies, the test might be more sensitive to detecting bias. The slope of the regression line (-0.03) suggests a very weak, near-flat line, indicating no clear trend between study size and effect size. However, the non-significant p-value (0.284) associated with the slope reinforces the uncertainty.

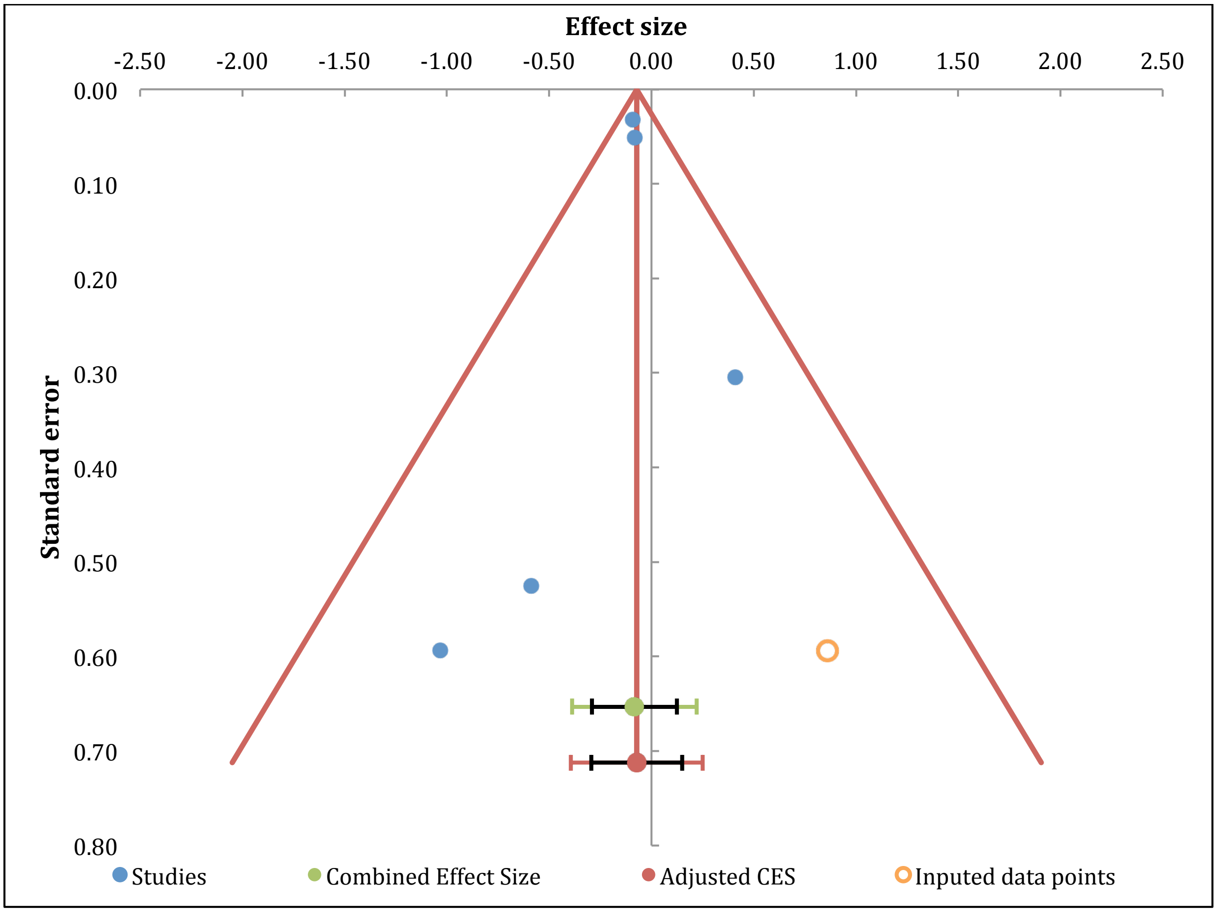

Figure-4. Any Serious Adverse events (Secondary outcome)

Egger Regression					t test	p-value
	Estimate	SE	CI LL	CI UL		
Intercept	-0.55	0.99	-3.29	2.18	-0.56	0.613
Slope	-0.02	0.14	-0.40	0.36		

The funnel plot visually depicted the relationship between study size (standard error) and effect size. Two studies fell along an adjusted Control Estimator's (CES) line, suggesting minimal bias. Two studies appeared above the funnel plot, potentially indicating an overestimation of the effect. Additionally, one data point with imputed data (represented by a green circle) fell on the positive side of the funnel plot. The Egger's test results themselves didn't show statistically significant evidence of publication bias (intercept: -0.55, p-value = 0.613). However, due to the small sample size (n = 5), we need to be cautious with this interpretation. A larger number of studies would provide a more robust test for detecting publication bias. The slope of the regression line (-0.02) suggests a very weak, if any, trend. There's no clear indication of smaller studies overestimating the effect. However, the non-significant p-value associated with the slope (0.613) reinforces the uncertainty surrounding this observation.

Table 2a. Cochrane risk of bias of the included Randomized controlled trials.

Studies	Primary Author	Study ID	Domain 1: Risk of bias arising from the randomization process (Selection bias)				Domain 2a: Risk of bias due to deviations from the intended interventions (effect of assignment to intervention) (Detection bias)								Domain 3: Missing outcome data (Attrition bias)					Domain 4: Risk of bias in measurement of the outcome (Reporting bias and appropriateness of the measurement method itself)						Domain 5: Risk of bias in selection of the reported result (Reporting bias)					Overall risk of bias
			1.1 Was the allocation sequence random?	1.2 Was the allocation sequence concealed until participants were enrolled and assigned to interventions?	1.3 Did baseline differences between intervention groups suggest a problem with the randomization process?	Risk-of-bias judgement	2.1. Were participants aware of their assigned intervention during the trial?	2.2. Were carers and people delivering the interventions aware of participants' assigned intervention during the trial?	2.3. If Y/PY/NI to 2.1 or 2.2: Were there deviations from the intended intervention that arose because of the trial context?	2.4 If Y/PY to 2.3: Were these deviations likely to have affected the outcome?	2.5. If Y/PY/NI to 2.4: Were these deviations from intended intervention balanced between groups?	2.6 Was an appropriate analysis used to estimate the effect of assignment to intervention?	2.7 If N/PN/NI to 2.6: Was there potential for a substantial impact (on the result) of the failure to analyse participants in the group to which they were randomized?	Risk-of-bias judgement	3.1 Were data for this outcome available for all, or nearly all, participants randomized?	3.2 If N/PN/NI to 3.1: Is there evidence that the result was not biased by missing outcome data?	3.3 If N/PN to 3.2: Could missingness in the outcome depend on its true value?	3.4 If Y/PY/NI to 3.3: Is it likely that missingness in the outcome depended on its true value?	Risk-of-bias judgement	4.1 Was the method of measuring the outcome inappropriate?	4.2 Could measurement or ascertainment of the outcome have differed between intervention groups?	4.3 If N/PN/NI to 4.1 and 4.2: Were outcome assessors aware of the intervention received by study participants?	4.4 If Y/PY/NI to 4.3: Could assessment of the outcome have been influenced by knowledge of intervention received?	4.5 If Y/PY/NI to 4.4: Is it likely that assessment of the outcome was influenced by knowledge of intervention received?	Risk-of-bias judgement	5.1 Were the data that produced this result analysed in accordance with a pre-specified analysis plan that was finalized before unblinded outcome data were available for analysis?	Is the numerical result being assessed likely to have been selected, on the basis of the results, from...	5.2. ... multiple eligible outcome measurements (e.g. scales, definitions, time points) within the outcome domain?	5.3 ... multiple eligible analyses of the data?	Risk-of-bias judgement	Risk-of-bias judgement
The SGLT2 inhibitor dapagliflozin in heart failure with preserved ejec-	PRESERVED-HF trial	Nassif *et al.*	Y	Y	N	Low	N	N	NA	NA	NA	Y	NA	Low	NA	NA	NA	NA	Low	N	N	N	NA	N	Low	Y	-	N	N	Low	Low

tion fraction: a multicenter randomized trial																															
Empagliflozin in Heart Failure with a Preserved Ejection Fraction: the EMPEROR-Preserved trial	EMPEROR-Preserved trial	Anker *et al.*	Y	Y	N	Low	N	N	NA	NA	NA	Y	NA	Low	NA	NA	NA	NA	Low	N	N	N	NA	N	Low	Y	-	N	N	Low	Low
Dapagliflozin and Cardiovascular Outcomes in Type 2 Diabetes	DECLARE–TIMI	Wiviott *et al.*	Y	Y	N	Low	N	N	NA	NA	NA	Y	NA	Low	NA	NA	NA	NA	Low	N	N	N	NA	N	Low	Y	-	N	N	Low	Low

Studies	Primary Author	Study ID	Domain 1: Risk of bias arising from the randomization process (Selection bias)				Domain 2a: Risk of bias due to deviations from the intended interventions (effect of assignment to intervention) (Detection bias)								Domain 3: Missing outcome data (Attrition bias)					Domain 4: Risk of bias in measurement of the outcome (Reporting bias and appropriateness of the measurement method itself)						Domain 5: Risk of bias in selection of the reported result (Reporting bias)					Overall risk of bias
Efficacy of Ertugliflozin on Heart Failure–Related Events in Patients With Type 2 Diabetes Mellitus and Established Atherosclerotic Cardiovascular Disease	VERTIS CV	Consentino *et al.*	Y	Y	N	Low	N	N	NA	NA	NA	Y	NA	Low	NA	NA	NA	NA	Low	N	N	N	NA	N	Low	Y	-	N	N	Low	Low
Dapagliflozin in Heart Failure	DELIVER	Solomon *et al.*	Y	Y	N	Low	N	N	NA	NA	NA	Y	NA	Low	NA	NA	NA	NA	Low	N	N	N	NA	N	Low	Y	-	N	N	Low	Low

with Mildly Reduced or Preserved Ejection Fraction																															
Effect of the Sodium-Glucose Cotransporter 2 Inhibitor Canagliflozin for Heart Failure With Preserved Ejection Fraction in Patients With Type 2 Diabetes.	CANONICAL	Ueda *et al.*	Y	PY	N	Low	PY	PY	NI	NA	NA	NI	NI	High	NA	NA	NA	NA	Low	N	N	PY	PY	N	High	Y	-	N	N	Low	High
Effects of canagliflozin in patients with type 2 diabetes and chronic heart failure: a randomized trial (CANDLE)	CANDLE	Tanaka *et al.*	Y	PY	N	Low	PY	PY	NI	NA	NA	NI	NI	High	NA	NA	NA	NA	Low	N	N	PY	PY	N	High	Y	-	N	N	Low	High

Abbreviations: Y: Yes; PY: Probably Yes; N: No; PN: Probably No; NI: No information.

Table 2b. Correlation of quality measures with estimates of treatment effects in meta-analyses of the included Randomized controlled trials (RCTs).

**Correlation of Quality Measures with Estimates of Treatment Effects in the Meta-analyses of RCTs**	**Nassif *et al.***	**Anker *et al.***	**Wiviott *et al.***	**Consentino *et al.***	**Solomon *et al.***	**Ueda *et al.***	**Tanaka *et al.***
RCT Name	PRESERVED-HF trial	EMPEROR-Preserved trial	DECLARE-TIMI	VERTIS CV	DELIVER	CANONICAL	CANDLE
Study question well-defined in introduction/methods	Yes	Yes	Yes	Yes	Yes	Yes	Yes
Study question well-defined anywhere in the article	Yes	Yes	Yes	Yes	Yes	Yes	Yes
Placebo control	Yes	Yes	Yes	Yes	Yes	No	No

Appropriate outcome studied	Yes	Yes	Yes	Yes	Yes	Yes	Yes
Multicenter study	Yes	Yes	Yes	Yes	Yes	Yes	Yes
Study country	USA	Germany	USA	USA	USA	Japan	Japan
Adequate selection criteria	Yes	Yes	Yes	Yes	Yes	Yes	Yes
Randomization methods described	Yes	Yes	Yes	Yes	Yes	Yes	Yes
Central randomization site	Yes	Yes	Yes	Yes	Yes	Yes	Yes
Allocation concealment	Yes	Yes	Yes	Yes	Yes	Yes	Yes
Patients blinded	Yes	Yes	Yes	Yes	Yes	No	No
Caregivers blinded	Yes	Yes	Yes	Yes	Yes	No	No
Outcome assessors blinded	No	Yes	Yes	Yes	Yes	No	Yes
Data analysts blinded	No	Yes	Yes	Yes	Yes	No	No
Double blinded	Yes	Yes	Yes	Yes	Yes	No	No
Vital statistical measures	Yes	Yes	Yes	Yes	Yes	Yes	Yes
Statistician author or acknowledged	No	No	No	No	No	No	Yes
Intention-to-treat analysis	Yes	Yes	Yes	Yes	Yes	No	No
Power calculation reported	Yes	Yes	Yes	Yes	Yes	No	Yes
Stopping rules described	Yes	Yes	Yes	Yes	Yes	No	Yes
Baseline characteristics reported	Yes	Yes	Yes	Yes	Yes	Yes	Yes
Groups similar at baseline	Yes	Yes	Yes	Yes	Yes	Yes	Yes
Confounders accounted for	N/D	N/D	Yes	Yes	Yes	N/D	N/D
Percentage dropouts	N/A	Yes	Yes	Yes	Yes	N/A	Yes
Reasons for dropout given	N/A	Yes	Yes	Yes	Yes	N/A	Yes
Findings support conclusion	Yes	Yes	Yes	Yes	Yes	Yes	Yes

Table 3. Certainty of the evidence (GRADE) profile at outcome level.

Patient or population: heart failure with mild plus preserved ejection Fraction
Intervention: SGLT2 Inhibitors
Comparison: placebo

Outcomes	**Anticipated absolute effects*** (95% CI)		Relative effect (95% CI)	№ of participants (studies)	Certainty of the evidence (GRADE)	Comments
	**Risk with placebo**	**Risk with SGLT2 Inhibitors**				
Hospitalization due to Heart Failure	71 per 1,000	**53 per 1,000** (49 to 58)	**OR 0.74** (0.67 to 0.81)	31057 (7 studies)	⊕⊕⊕ Moderate	Downgraded for observed risk of bias, publication bias uncertainty, and observed overestimation of the effect in Egger's regression analysis in included studies and upgraded for very large magnitude of effect and less heterogeneity.

Patient or population: heart failure with mild plus preserved ejection Fraction
Intervention: SGLT2 Inhibitors
Comparison: placebo

Outcomes	**Anticipated absolute effects*** (95% CI)		Relative effect (95% CI)	№ of participants (studies)	Certainty of the evidence (GRADE)	Comments
	**Risk with placebo**	**Risk with SGLT2 Inhibitors**				
Hospitalization Due to heart Failure after excluding LVEF <50%	110 per 1,000	**81 per 1,000** (70 to 95)	**OR 0.72** (0.61 to 0.85)	6627 (4 studies)	⨁⨁⨁ Moderate	Downgraded for observed risk of bias, publication bias uncertainty, and observed overestimation of the effect in Egger's regression analysis in included studies and upgraded for very large magnitude of effect and less heterogeneity.
Death due to cardiovascular causes	51 per 1,000	**48 per 1,000** (43 to 52)	**OR 0.92** (0.83 to 1.02)	30500 (5 studies)	⨁⨁⨁ Moderate	Downgraded for observed risk of bias, publication bias uncertainty, and observed overestimation of the effect in Egger's regression analysis in included studies and upgraded for very large magnitude of effect and less heterogeneity.
All-cause mortality	101 per 1,000	**96 per 1,000** (89 to 103)	**OR 0.94** (0.87 to 1.02)	31057 (7 studies)	⨁⨁⨁ Moderate	Downgraded for observed risk of bias, publication bias uncertainty, and observed overestimation of the effect in Egger's regression analysis in included studies and upgraded for very large magnitude of effect and less heterogeneity.
Any Serious Adverse events	380 per 1,000	**360 per 1,000** (337 to 384)	**OR 0.92** (0.83 to 1.02)	24062 (5 studies)	⨁⨁⨁ Moderate	Downgraded for observed risk of bias, publication bias uncertainty, and observed overestimation of the effect in Egger's regression analysis in included studies and upgraded for very large magnitude of effect and less heterogeneity.

***The risk in the intervention group** (and its 95% confidence interval) is based on the assumed risk in the comparison group and the **relative effect** of the intervention (and its 95% CI).

CI: confidence interval; **OR:** odds ratio

GRADE Working Group grades of evidence
High certainty: we are very confident that the true effect lies close to that of the estimate of the effect.
Moderate certainty: we are moderately confident in the effect estimate: the true effect is likely to be close to the estimate of the effect, but there is a possibility that it is substantially different.
Low certainty: our confidence in the effect estimate is limited: the true effect may be substantially different from the estimate of the effect.
Very low certainty: we have very little confidence in the effect estimate: the true effect is likely to be substantially different from the estimate of effect.

Section and Topic	Item #	Checklist item	Location where item is reported
**TITLE**			
Title	1	Identify the report as a systematic review.	1
**ABSTRACT**			
Abstract	2	See the PRISMA 2020 for Abstracts checklist.	2
**INTRODUCTION**			
Rationale	3	Describe the rationale for the review in the context of existing knowledge.	3
Objectives	4	Provide an explicit statement of the objective(s) or question(s) the review addresses.	4
**METHODS**			
Eligibility criteria	5	Specify the inclusion and exclusion criteria for the review and how studies were grouped for the syntheses.	5-6
Information sources	6	Specify all databases, registers, websites, organisations, reference lists and other sources searched or consulted to identify studies. Specify the date when each source was last searched or consulted.	5
Search strategy	7	Present the full search strategies for all databases, registers and websites, including any filters and limits used.	5
Selection process	8	Specify the methods used to decide whether a study met the inclusion criteria of the review, including how many reviewers screened each record and each report retrieved, whether they worked independently, and if applicable, details of automation tools used in the process.	5-6
Data collection process	9	Specify the methods used to collect data from reports, including how many reviewers collected data from each report, whether they worked independently, any processes for obtaining or confirming data from study investigators, and if applicable, details of automation tools used in the process.	5-8
Data items	10a	List and define all outcomes for which data were sought. Specify whether all results that were compatible with each outcome domain in each study were sought (e.g. for all measures, time points, analyses), and if not, the methods used to decide which results to collect.	6
	10b	List and define all other variables for which data were sought (e.g. participant and intervention characteristics, funding sources). Describe any assumptions made about any missing or unclear information.	6
Study risk of bias assessment	11	Specify the methods used to assess risk of bias in the included studies, including details of the tool(s) used, how many reviewers assessed each study and whether they worked independently, and if applicable, details of automation tools used in the process.	8
Effect measures	12	Specify for each outcome the effect measure(s) (e.g. risk ratio, mean difference) used in the synthesis or presentation of results.	6
Synthesis methods	13a	Describe the processes used to decide which studies were eligible for each synthesis (e.g. tabulating the study intervention characteristics and comparing against the planned groups for each synthesis (item #5)).	6-8
	13b	Describe any methods required to prepare the data for presentation or synthesis, such as handling of missing summary statistics, or data conversions.	6-8
	13c	Describe any methods used to tabulate or visually display results of individual studies and syntheses.	6-8
	13d	Describe any methods used to synthesize results and provide a rationale for the choice(s). If meta-analysis was performed, describe the model(s), method(s) to identify the presence and extent of statistical heterogeneity, and software package(s) used.	6-8

Section and Topic	Item #	Checklist item	Location where item is reported
	13e	Describe any methods used to explore possible causes of heterogeneity among study results (e.g. subgroup analysis, meta-regression).	6-8
	13f	Describe any sensitivity analyses conducted to assess robustness of the synthesized results.	6-8
Reporting bias assessment	14	Describe any methods used to assess risk of bias due to missing results in a synthesis (arising from reporting biases).	8
Certainty assess-	15	Describe any methods used to assess certainty (or confidence) in the body of evidence for an outcome.	8

sess-ment			
**RESULTS**			
Study selection	16a	Describe the results of the search and selection process, from the number of records identified in the search to the number of studies included in the review, ideally using a flow diagram.	8-9
	16b	Cite studies that might appear to meet the inclusion criteria, but which were excluded, and explain why they were excluded.	8-9
Study characteristics	17	Cite each included study and present its characteristics.	9
Risk of bias in studies	18	Present assessments of risk of bias for each included study.	12
Results of individual studies	19	For all outcomes, present, for each study: (a) summary statistics for each group (where appropriate) and (b) an effect estimate and its precision (e.g. confidence/credible interval), ideally using structured tables or plots.	9-11
Results of syntheses	20a	For each synthesis, briefly summarise the characteristics and risk of bias among contributing studies.	12
	20b	Present results of all statistical syntheses conducted. If meta-analysis was done, present for each the summary estimate and its precision (e.g. confidence/credible interval) and measures of statistical heterogeneity. If comparing groups, describe the direction of the effect.	9-11
	20c	Present results of all investigations of possible causes of heterogeneity among study results.	9-11
	20d	Present results of all sensitivity analyses conducted to assess the robustness of the synthesized results.	9-11
Reporting biases	21	Present assessments of risk of bias due to missing results (arising from reporting biases) for each synthesis assessed.	12
Certainty of evidence	22	Present assessments of certainty (or confidence) in the body of evidence for each outcome assessed.	12
**DISCUSSION**			
Discussion	23a	Provide a general interpretation of the results in the context of other evidence.	12-13
	23b	Discuss any limitations of the evidence included in the review.	16-17
	23c	Discuss any limitations of the review processes used.	16-17
	23d	Discuss implications of the results for practice, policy, and future research.	16
**OTHER INFORMATION**			
**Section and Topic**	**Item #**	**Checklist item**	**Location where item is reported**
Registration and protocol	24a	Provide registration information for the review, including register name and registration number, or state that the review was not registered.	2, 5
	24b	Indicate where the review protocol can be accessed, or state that a protocol was not prepared.	NA
	24c	Describe and explain any amendments to information provided at registration or in the protocol.	18
Support	25	Describe sources of financial or non-financial support for the review, and the role of the funders or sponsors in the review.	18
Competing interests	26	Declare any competing interests of review authors.	18
Availability of data, code and other materials	27	Report which of the following are publicly available and where they can be found: template data collection forms; data extracted from included studies; data used for all analyses; analytic code; any other materials used in the review.	18

From: Page MJ, McKenzie JE, Bossuyt PM, Boutron I, Hoffmann TC, Mulrow CD, et al. The PRISMA 2020 statement: an updated guideline for reporting systematic reviews. BMJ 2021;372:n71. doi: 10.1136/ bmj.n71

For more information, visit: http://www.prisma-statement.org/
